# Supplementary material for: Methanol-driven esterification of volatile short-chain fatty acids in thermal desorption-based analysis
Source: Commun Chem. 2026 Apr 1;9:189. doi: 10.1038/s42004-026-01998-5 (PMC13213016; doi:10.1038/s42004-026-01998-5)
Supplement: Supplementary file 2 — Description of Additional Supplementary Files [file 42004_2026_1998_MOESM2_ESM.pdf]

Description of Additional Supplementary Files:

File: Supplementary Data

Description: Numerical Source Data of all Figures 1-4
